# Improved Dynamic Event-Triggered Robust Control for Flexible Robotic Arm Systems with Semi-Markov Jump Process

**DOI:** 10.3390/s23125523

**Published:** 2023-06-12

**Authors:** Huiyan Zhang, Zixian Chen, Wengang Ao, Peng Shi

**Affiliations:** 1 National Research Base of Intelligent Manufacturing Service, Chongqing Technology and Business University, Chongqing 400067, China; huiyanzhang@ctbu.edu.cn (H.Z.);; 2 National Engineering Laboratory for Industrial Big-Data Application Technology, Chongqing Innovation Center of Industrial Big-Data Co., Ltd., Chongqing 400707, China; 3 School of Mechanical Engineering Chongqing Technology and Business University, Chongqing 400067, China; zixianchen133@gmail.com; 4 School of Electrical and Mechanical Engineering, The University of Adelaide, Adelaide, SA 5005, Australia; 5 School of Information Science and Technology, Fujian University of Technology, Fuzhou 350118, China

**Keywords:** flexible robotic arm, dynamic event-triggered scheme, phase-type semi-Markov chain, robust control, DoS attacks

## Abstract

In this paper, we investigate the problem of a dynamic event-triggered robust controller design for flexible robotic arm systems with continuous-time phase-type semi-Markov jump process. In particular, the change in moment of inertia is first considered in the flexible robotic arm system, which is necessary for ensuring the security and stability control of special robots employed under special circumstances, such as surgical robots and assisted-living robots which have strict lightweight requirements. To handle this problem, a semi-Markov chain is conducted to model this process. Furthermore, the dynamic event-triggered scheme is used to solve the problem of limited bandwidth in the network transmission environment, while considering the impact of DoS attacks. With regard to the challenging circumstances and negative elements previously mentioned, the adequate criteria for the existence of the resilient $H_{\infty }$ controller are obtained using the Lyapunov function approach, and the controller gains, Lyapunov parameters and event-triggered parameters are co-designed. Finally, the effectiveness of the designed controller is demonstrated via numerical simulation using the LMI toolbox in MATLAB.

## 1. Introduction

With the development of information technology, physical systems in practical applications are often composed of a large number of interacting elements, which are called complex systems, such as robotic arm control [1], power system [2], and multi-agent system [3,4]. As a special class of complex systems, Markov jump systems (MJSs) are widely studied by scholars for their superior modeling capacity to deal with unexpected changes, which may include environmental disruptions, random component mistakes, or even human influences during regular operation [5,6]. The authors in [7,8,9] reviewed recent advancements in modeling, stability analysis, filter design and sliding model control of MJSs. A new controller was proposed to ensure the stabilization of generalized MJSs in [10] to solve the random stabilization problem. Zhuang et al. [11] considered both the stability analysis and stabilization of generalized Markov jump time-delay systems, with the relevant state-feedback controller derived by means of LMI.

However, there exists a limitation in practical applications whereby the sojourn time of the Markov chain follows an exponential probability distribution, that is, results on MJSs are unfortunately conservative for the constant transition rates as a memoryless property of the exponential distribution. To solve this problem, a new model referred to as semi-Markov chain with less conservative sojourn time was proposed in [12,13,14]. Phase-type semi-Markov jump systems, which present a special class of semi-Markov jump systems, are increasingly attracting the attention of researchers, since the corresponding sojourn time obeys the characteristics of PH distribution. The problem of positive $L_{1}$ filters designed for nonlinear stochastic-switched systems, which are sensitive to the phase-type semi-Markov jump process, was explored in [15]. The work in [16] was extended to solve the problem of positive $L_{1}$ fuzzy filters designed for phase-type semi-Markov jump singular systems, which can be translated into a corresponding Markov jump singular system using an equivalent transformation [15].

Benefiting from the technology developed for the rapid growth of network communications, NCSs (network control systems) have gradually replaced physical transmission media with their unique advantages: low construction cost, small required space, low physical failure rate [17]. These advantages have recently attracted significant research interest from scholars. However, the introduction of network control systems has also created new difficulties for networked system analysis and synthesis [18]. We consider the inevitable shortcomings (packet mis-order, transmission delay, and data packet loss) of the network itself when designing a controller. On one hand, cyber-attacks will reduce the stability of networked systems. The basic goal of deception assaults is to alter the true signal while introducing fake signals. On the other hand, the occurrence of a time-delay phenomenon also causes instability or oscillation of the system, thus increasing the difficulty of system stability analysis. For a type of discrete Markovian jump system that suffers from random packet loss and time-varying delays, Zhao et al. addressed the finite time $H_{\infty }$ robust control issue in their work [19]. Regarding the uncertain discrete Markov jump time-varying delay polyhedron system, Shi et al. [20] used Lyapunov functional theory and convex polyhedron technique, to provide the random admissibility of the generalized model error augment system. In addition, the actual system may suffer from sudden changes in internal structure or the external environment.

In 1999, Arzen K proposed the concept of an event trigger for the first time in [21], which subsequently attracted wide attention due to its superiority in network communication. In 2013, Dong Y built an event flip-flop and NCSs into a unified time-delay system model [22]. Compared to a static event trigger, by introducing an internal function to reduce the conservatism of the trigger condition in the form of a dynamic event trigger, this internal function can store part of the system information which allows further reduction in resource consumption under the premise of safety and control. In [23], for linear time-invariant systems, a unique dynamic event-triggered control mechanism is described. By deriving criteria for this control strategy that provide asymptotic stability and $L_{2}$ stability, the triggering parameters and feedback gain may be jointly constructed. Dynamic event-triggering mechanism is the suggested name for this class in [24] due to the introduction of an internal dynamic variable being a defining feature of it. In [25], multiple types of network attack signals are considered in multi-agent systems with multiple cyber-attacks. According to Lyapunov stability theory and the linear matrix inequalities (LMIs) method, exponentially mean-square finite-time stability is derived from these systems. The annular finite-time bounded and adaptive event-triggered control issues of networked switched systems with deception attacks are examined in [26]; however, no concrete engineering application is given in the paper. To the extent of the authors’ knowledge, however, there is scarce literature on the robust properties of semi-Markov jump systems with a dynamic event-triggering mechanism.

Due to the project needs and the hardware situation of the laboratory, a flexible robot arm is used as the research object. The flexible robotic arm systems proposed in this paper takes the change in moment of inertia into account for the first time. This is a necessary requirement for ensuring the safety and stability control of special robots that are applied under certain circumstances, e.g., surgical robots and assisted-living robots which have strict lightweight requirements. The main contributions of this paper are as follows: (1) We consider the $H_{\infty }$ performance of the flexible robotic arm with phase-type semi-Markov process; (2) The necessary conditions are found for robust controllers to exist by using the Lyapunov function method, furthermore, the controller and the dynamic event-triggering scheme are co-designed; (3) A simulation is conducted on the flexible robotic arm using the LMI toolbox in MATLAB, to estimate the usefulness of the designed controller gain with the comparison diagram of the time-trigger mechanism and the dynamic event-trigger mechanism.

The remainder of this paper is structured as follows. Section 2 provides a description of the system and a statement of the problem, along with some fundamental ideas and lemmas. Principal conclusions from a stability analysis and time-/event-triggered robust controller for flexible robotic arm systems with semi-Markov jump process are rendered in Section 3. In Section 4, a numerical simulation method is used to demonstrate the functional operation of the suggested controller design process.

Notation. There are various notations used throughout this paper. In the upper-right section of the matrix, −1 and *T* represent the inverse and transpose of matrix, respectively, and ∗ in the symmetric matrix denotes the omitted term, and diag{·} means a block diagonal matrix. Notation $0_{n\times m}$ represents the zero matrix of dimension n×m. Furthermore, the 2–norm of vectors in this paper are represented by ${\left||*|\right|}_{2}$, and $R^{0+}$ denotes the set of positive real numbers. Finally, E{·} represents the mathematical expectation. The remaining notations are less general in nature and are shown after their respective formula.

## 2. System Description and Preliminaries

### 2.1. Physical Model of Flexible Robotic Arm

**Figure 1:**
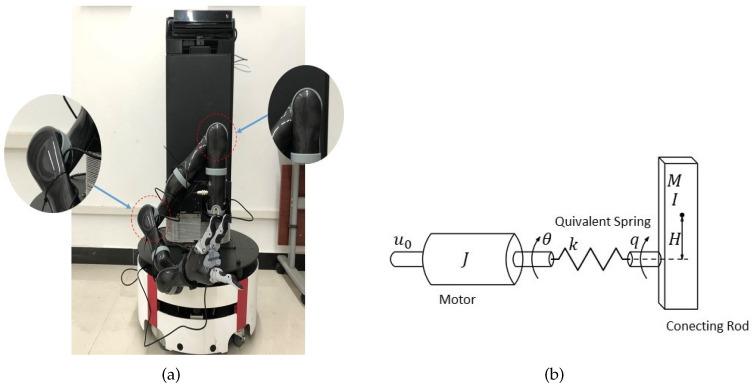
Model Overview. (**a**) Physical model of flexible robotic arm; (**b**) Dynamical model of flexible joint.

In this paper, a single link manipulator driven by DC motor with flexible joint is used as the controlled object (as shown in Figure 1a). The dynamic characteristics of a flexible joint can be approximated by a linear torsion spring with an elastic coefficient *k*. Inspired by [27], the structure is shown in Figure 1b. The ideal dynamic equations of the flexible robotic arm systems are given by

(1) $$\begin{array}{ccc} I\ddot{q}\left(t\right)+MgH\sin q\left(t\right) & = & k\left(\theta \left(t\right)-q\left(t\right)\right)+\mu_{1}\left(t\right)\\ J\ddot{\theta }\left(t\right)+k\left(\theta \left(t\right)-q\left(t\right)\right) & = & u_{0}\left(t\right)+\mu_{2}\left(t\right) \end{array}$$

where θ(t) is the rotation angle, *J* is the equivalent moment, $\mu_{1}\left(t\right)$ represents external disturbance of motor; q(t), *I*, $\mu_{2}\left(t\right)$ represent the same parameters of the connecting rod. *k* represents the equivalent spring elastic coefficient; $u_{0}\left(t\right)$ is the input torque of the motor; *M* is the weight of the connecting rod; *H* is the center of mass and the distance between the axis of rotation, the acceleration of gravity is represented by *g*.

Let us define the following state variables:

(2) $$\begin{array}{c} x_{1}\left(t\right)=q\left(t\right),\;x_{2}\left(t\right)=\dot{q}\left(t\right),\;x_{3}\left(t\right)=\theta \left(t\right),\;x_{4}\left(t\right)=\dot{\theta }\left(t\right) \end{array}$$

where $x_{1}\left(t\right)$ denotes the rotation angle of the DC motor, $x_{2}\left(t\right)$ is the angular velocity of the motor, $x_{3}\left(t\right)$ is the rotation angle of connecting rod, and $x_{4}\left(t\right)$ denotes the angular velocity of the connecting rod.

**Assumption 1.**  *The nonlinear term sinq(t) is presented in the dynamic equation mentioned above. We make the assumption that $\sin q\left(t\right)=q\left(t\right),\;q\left(t\right)\in \left(-\frac{\pi }{2},\frac{\pi }{2}\right)$ to remove the nonlinear term’s influence. In other words, the connecting rod’s accessible range is partially constrained.*

**Figure 2:**
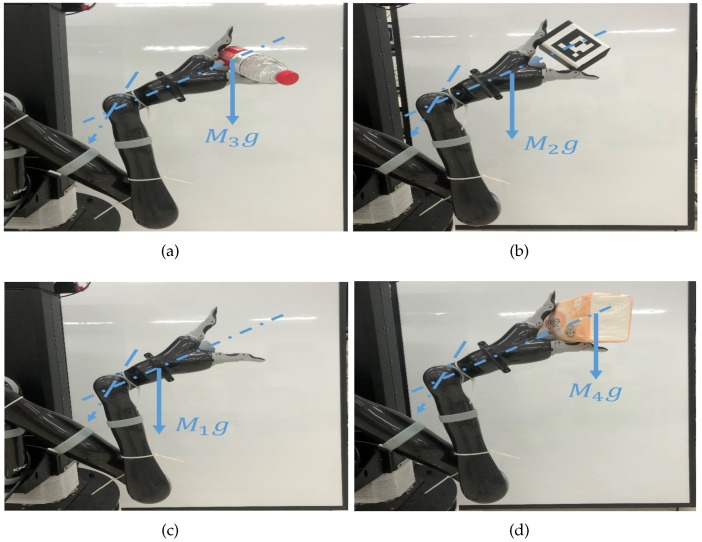
Moment of inertia under different working conditions. (**a**) $I_{1}$ of State 1; (**b**) $I_{1}$ of State 2; (**c**) $I_{1}$ of State 3; (**d**) $I_{1}$ of State 4.

It is well known that the moment of inertia depends on: (a) the shape of the object, (b) the position of the axis of rotation, and (c) the distribution of mass. When grasping objects of different masses, where (a,b) remain unchanged and (c) changes with the end-effector grasping different objects, the moment of inertia of the connecting rod has different modes in Figure 2. A flexible joint manipulator system is a complex system with continuous time and discrete state.

Take into consideration the subsequent continuous-time stochastic systems over a probability space ($℧,F,P_{r}$) with Semi-Markov properties, add the dynamical equation as shown below.

(3) $$\begin{array}{c} \left\{\begin{array}{ccc} \dot{x}\left(t\right) & = & \hat{A}\left(t,\hat{r}\left(t\right)\right)x\left(t\right)+{\hat{B}}_{1}\left(\hat{r}\left(t\right)\right)\omega \left(t\right)+B_{2}u\left(t\right),\\ z(t) & = & Cx(t), \end{array}\right. \end{array}$$

where $x\left(t\right)={\left[x_{1}\left(t\right)\;\;x_{2}\left(t\right)\;\;x_{3}\left(t\right)\;\;x_{4}\left(t\right)\right]}^{T}$, ω(t) means the external disturbance and $\omega^{T}\left(t\right)\omega \left(t\right)$≤I, u(t) represents the input signal of the closed-loop system, and

$$\begin{array}{ccc} \hat{A}\left(t,\hat{r}\left(t\right)\right) & = & \left[\begin{array}{cccc} 0 & 1 & 0 & 0\\ -\frac{MgH+k}{I(t,\hat{r}\left(t\right))} & 0 & \frac{k}{I(t,\hat{r}\left(t\right))} & 0\\ 0 & 0 & 0 & 1\\ \frac{k}{J} & 0 & -\frac{k}{J} & 0 \end{array}\right],\;{\hat{B}}_{1}\left(t,\hat{r}\left(t\right)\right)=\left[\begin{array}{c} 0\\ \frac{1}{I(t,\hat{r}\left(t\right))}\\ 0\\ \frac{1}{J} \end{array}\right],\;B_{2}=\left[\begin{array}{c} 0\\ 0\\ 0\\ \frac{1}{J} \end{array}\right], \end{array}$$

and C=[1000]. $\hat{r}\left(t\right)$ represents a finite state semi-Markov jump process, which accepts discrete values inside the specified finite set {1,2,⋯,m+1}, the state m+1 is absorbing and other states are transient. Abbreviated symbols, ${\hat{A}}_{i}$ for $\hat{A}\left(t,r\left(t\right)\right)$, ${\hat{B}}_{1i}$ for ${\hat{B}}_{1}\left(t,r\left(t\right)\right)$. The infinitesimal generator is

(4) $$\begin{array}{ccc} \Xi & = & \left[\begin{array}{cc} T_{m\times m} & T_{m\times 1}^{0}\\ T_{1\times m} & 0 \end{array}\right] \end{array}$$

where matrix $T_{m\times m}=\left(T_{ij}\right)$ satisfies $T_{ij}<0,T_{ij}\geq 0,i\neq j,$ the non-negative column vector $T^{0}$ meets $Te+T^{0}=0$. While (a,m+1) is represented as the initial distribution vector, where $a=\left(a_{1},a_{2},\cdots ,a_{m}\right),ae+a_{m+1}=1,$ and *e* stands for an m-dimensional column vector made up only of 1.

Before moving on, let us review the following fundamental claims and definitions:

**Proposition 1.**  *The rate at which $\hat{r}\left(t\right)$ is absorbed in $\hat{r}\left(t\right)$ is distributed as*

(5) $$\begin{array}{ccc} F(t) & = & 1-a\exp(Tt)e,\;t\geq 0. \end{array}$$

**Definition 1.**  *The phase of the distribution F(·) at time t is the state that $\hat{r}\left(t\right)$ reaches at that instant. Its representation of order m is (a,e), and the distribution F(·) described in version (5) on [0,∞) is known as a phase-type (PH) distribution.*

If G is a finite set, then, Markov chain is the denumerable phase semi-Markov process, where $F_{i}\left(t\right)\left(i\in G\right)$ has a negative exponential distribution. However, the characteristic of Markov chain that the sojourn time obey negative exponential distribution can be overcome by a denumerable phase semi-Markov process. The main issue is whether or not the denumerable phase semi-Markov process can be changed into a Markov chain. Moreover, a finite Markov chain can be created from a finite phase semi-Markov process. The remainder of this paragraph will provide evidence supporting the validity of the aforementioned statement.

Let $\hat{r}\left(t\right)$ be a denumberable phase semi-Markov process on the state space G. Denote the *n*th jump point of process $\hat{r}\left(t\right)$ by $\iota_{n}\left(n=0,1,2,\cdots \right)$, where $\iota_{0}\equiv 0<\iota_{1}<\iota_{2},\cdots ,<\iota_{n},\cdots$. Let $\left(a^{i},T^{i}\right)\left(i\in G\right)$ stand for the representation of $F_{i}\left(t\right)$ in the $m^{i}$ order, where

$$\begin{array}{ccc} F_{i}\left(t\right) & = & P\left(\iota_{n+1}-\iota_{n}\leq t|\hat{r}\left(\iota_{n}\right)=i\right)\;\left(i\in G\right),\\ a^{i} & = & \left(a_{1}^{i},a_{2}^{i},\cdots ,a_{m^{i}}^{i}\right),\;T_{i}=\left(T_{jk}^{i},j,k\in G\right). \end{array}$$

Let

$$\begin{array}{ccc} \lambda_{ij} & = & P\left(\hat{r}\left(\iota_{n+1}\right)=j|\hat{r}\left(\iota_{n}=i\right)\right)\;\left(i,j\in G\right), \end{array}$$

(6) $$\begin{array}{ccc} P & = & (\lambda_{ij},i,j\in G), \end{array}$$

(7) $$\begin{array}{ccc} \left(a,T\right) & = & \{\left(a^{i},T^{i}\right),\;i\in G\}. \end{array}$$

It is clear that only {(P,(a,T))} can predict the probability distribution of $\hat{r}\left(t\right)$.

**Definition 2.**  *The pair of the semi-Markov process $\hat{r}\left(t\right)$ is referred to as {P,(a,T)}. Define χ(t)= the phase of $F_{\hat{r}\left(t\right)}\left(\cdot \right)$ at time $t-\iota_{n}$ for every $n\left(n=0,1,\cdots \right),\iota_{n}\leq t<\iota_{n+1}$. For any i∈G, we define*

$$\begin{array}{ccc} T_{j}^{i,0} & = & 1-\sum_{k=1}^{m(i)}T_{jk}^{i}\;\left(j=1,2,\cdots ,m^{i}\right),\\ U & = & \{\left(i,k^{i}\right)|i\in G,k^{i}=1,2,\cdots ,m^{i}\}. \end{array}$$

We can readily obtain the following conclusions from the aforementioned analysis.

**Lemma 1.**  *Markov chain $Z\left(t\right)=(\hat{r}\left(t\right),\chi \left(t\right))$ has state space U. The pair of $\hat{r}\left(t\right)$ given by P,(a,T) determines the infinitesimal generator of Z(t) exclusively, as shown in the following*

$$\begin{array}{c} \left\{\begin{array}{ccc} \beta_{\left(i,k^{i}\right)\left(i,k^{i}\right)} & = & T_{k^{i}k^{i}}^{i},\;\left(i,k^{i}\in U\right),\\ \beta_{\left(i,k^{i}\right)\left(i,{\bar{k}}^{i}\right)} & = & T_{k^{i}{\bar{k}}^{i}}^{i},\;k^{i}\neq {\bar{k}}^{i},\left(i,k^{i}\right)\in U\;and\;\left(i,{\bar{k}}^{i}\right)\in U,\\ \beta_{\left(i,k^{i}\right)\left(j,k^{j}\right)} & = & \lambda_{ij}T_{k^{i}}^{\left(i,0\right)}a_{k^{j}}^{j},\;i\neq j,\left(i,k^{i}\right)\in U\;and\;\left(j,k^{j}\right)\in U. \end{array}\right. \end{array}$$

Suppose that U has $s=\sum_{i\in G}m^{i}$ elements, which results in *s* elements in the state space of Z(t). The *s* elements are numbered using the following procedure: the number of (i,k) by $\sum_{r=1}^{i-1}m^{r}+k\left(1\leq k\leq m^{i}\right)$. Adding the letter ψ to this transformation as well, and one has

(8) $$\begin{array}{ccc} \psi ((i,k)) & = & \sum_{r=1}^{i-1}m^{r}+k\;\left(i\in G,1\leq k\leq m^{i}\right). \end{array}$$

Furthermore, we define

(9) $$\begin{array}{ccc} \upsilon_{\psi \left(\left(i,k\right)\right)\psi \left(\left(i^{\prime },k^{\prime }\right)\right)} & = & \beta_{\left(i,k\right)\left(i^{\prime },k^{\prime }\right)}, \end{array}$$

$$\begin{array}{c} r(t)=\psi (Z(t)). \end{array}$$

The infinitesimal generator $\Xi =(\upsilon_{im},1\leq i,m\leq s)$ with a state space S={1,2,⋯,s} make r(t) be a Markov chain. Above all, the associated Markov chain of $\hat{r}\left(t\right)$ is the name given by the Markov chain r(t). The latest transition probability matrix satisfies that

(10) $$\begin{array}{c} P_{r}\left\{r\left(t+\delta \right)=j|r\left(t\right)=i\right\}=\left\{\begin{array}{cc} \pi_{ij}\delta +o\left(\delta \right), & i\neq j\\ 1+\pi_{ii}\delta +o\left(\delta \right), & i=j \end{array}\right. \end{array}$$

where $\pi_{ij}\geq 0$(i≠j) and $\sum_{j=1,i\neq j}^{s}\pi_{ij}=-\pi_{ii}$(i∈S) and o(δ)/δ→0 for δ>0. When δ→0, $r_{0}\in S$ is the initial condition for the continuous state.

### 2.2. Network Control Based on Dynamic Event-Triggered Scheme

**Figure 3:**
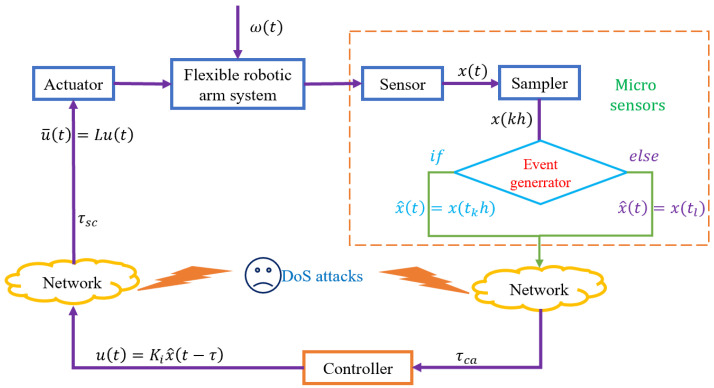
Static event-triggered control model.

**Figure 4:**
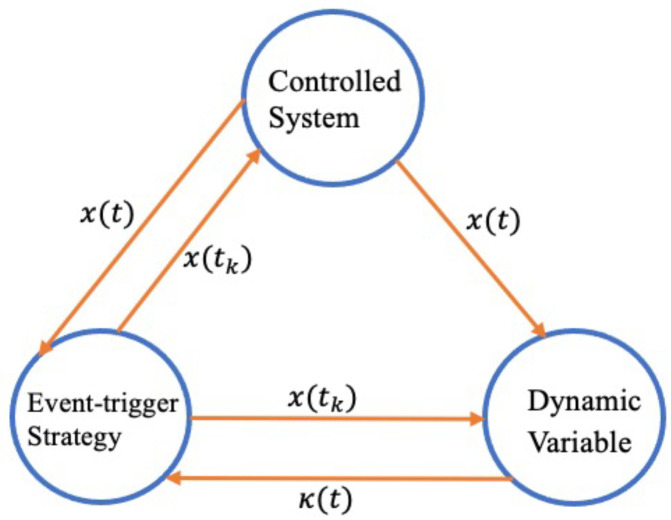
Dynamic event-triggered control model.

Benefit from the rapid development and wide application of network communication technology, the combination of control system and network transmission has attracting more and more attentions of scholars [28,29]. The traditional time-triggered/periodic-triggered control scheme has the shortcoming of wasting network resources (CPU for computing) which information are less important, the static event-triggered control in Figure 3 is introduced to solve this problem, which greatly alleviates the pressure of network bandwidth and reduces the waste of communication resources while implementing effective control. In this article, a dynamic event-triggered scheme in Figure 4 is proposed to further save the network bandwidth.

An intelligent sensor is composed of a sensor, sampler, event generator and memory. The sensor is responsible for collecting the sensitive information of the controlled object, and the sampler collects the continuous signals transmitted by the sensor at a fixed period. The event generator screens the received sampled data according to the preset event trigger conditions, and the sampled signals that meet the trigger conditions will be sent to the controller, otherwise they will not be sent. Sampling sequence $H_{1}=\left\{0,h,2h,\cdots ,kh\right\},k\in \left\{0,1,2,3,\cdots \right\}$, the time series of successful signal release is $H_{2}=\left\{t_{1},t_{2},t_{3},\cdots ,t_{k}\right\},k\in \left\{0,1,2,3,\cdots \right\}$. Clearly $H_{2}\in H_{1}$, the real control signal u(t) for the controller is provided by

(11) $$\begin{array}{ccc} u(t) & = & K_{i}x\left(t_{k}\right),\;t\in \left[t_{k},t_{k+1}\right), \end{array}$$

For $t\in \left[t_{k}+\tau_{t_{k}},t_{k+1}+\tau_{t_{k+1}}\right]$, motivated by [30], we can easily prove that there must be a positive integer $d_{M}\left(\geq 1\right)$ such that

$$\begin{array}{c} t_{k}+d_{M}h+\bar{\tau }<r_{k+1}+\tau_{r_{k+1}}\leq t_{k}+\left(d_{M}+1\right)h+\bar{\tau } \end{array}$$

$\left[t_{k}+\tau_{t_{k}},t_{k+1}+\tau_{t_{k+1}}\right]$ is divided into the following $d_{M}+1$ subintervals, where $t_{k}^{i}=t_{k}+ih,i=0,1,2,\cdots ,d_{M}$, we define

(12) $$\begin{array}{c} \left\{\begin{array}{ccc} {∆}_{0} & = & \left[t_{k}+\tau_{t_{k}},t_{k}^{1}+\bar{\tau }\right]\\ {∆}_{i} & = & \left[t_{k}^{i}+\bar{\tau },t_{k}^{i}+h+\bar{\tau }\right],\;i=1,2,\cdots ,d_{M}-1\\ {∆}_{d_{M}} & = & \left[t_{k}^{d_{M}}+\bar{\tau },t_{k+1}h+\tau_{t_{k+1}}\right] \end{array}\right. \end{array}$$

easy to know $\left[t_{k}+\tau_{t_{k}},t_{k+1}+\tau_{t_{k+1}}\right]=\cup_{i=0}^{d_{M}}{∆}_{i}$. Define a function τ(t):

(13) $$\begin{array}{c} \tau \left(t\right)=t-t_{k}^{i},t\in {∆}_{i},i=0,1,2,\cdots ,d_{M} \end{array}$$

According to the above equation:

(14) $$\begin{array}{c} \left\{\begin{array}{cc} \tau_{t_{k}}\leq \tau \left(t\right)\leq h+\bar{\tau } & t\in {∆}_{0}\\ \tau_{t_{k}}\leq \bar{\tau }\leq \tau \left(t\right)\leq h+\bar{\tau } & t\in {∆}_{i}\\ \tau_{t_{k}}\leq \bar{\tau }\leq \tau \left(t\right)\leq h+\bar{\tau } & t\in {∆}_{d_{M}} \end{array}\right. \end{array}$$

when $t\in \left[t_{k}+\tau_{t_{k}},t_{k+1}+\tau_{t_{k+1}}\right],\tau \left(t\right)\in \left[\tau_{1},\tau_{2}\right],\;\mathrm{where}\;\tau_{2}=h+\bar{\tau }$. Furthermore, let us define an error vector as following:

(15) $$\begin{array}{c} e\left(t\right)=\left\{\begin{array}{cc} 0, & t\in {∆}_{0},\\ x\left(t_{k}^{i}\right)-x\left(t_{k}\right), & t\in {∆}_{i},i=1,2,\cdots ,d_{M}-1,\\ x\left(t_{k}^{{∆}_{M}}\right)-x\left(t_{k}\right), & t\in {∆}_{M}. \end{array}\right. \end{array}$$

Then, dynamic event-triggered conditions will be written as follows.

(16) $$\begin{array}{c} \varrho \kappa \left(t\right)+\sigma x^{T}\left(t-\tau \left(t\right)\right)WX\left(t-\tau \left(t\right)\right)-e^{T}\left(t\right)We\left(t\right)\leq 0 \end{array}$$

where $\sigma \in \left(0,1\right),\varrho \in R_{0}^{+}$ are parameters to be designed; W>0 is an undetermined matrix with appropriate dimensions.

Furthermore, the internal variable κ(t) that fulfills the following differential equation is

(17) $$\begin{array}{c} \left\{\begin{array}{ccc} \dot{\kappa }\left(t\right) & = & -\varsigma \left(\kappa \left(t\right)\right)-e^{T}\left(t\right)We\left(t\right)+\sigma x^{T}\left(t-\tau \left(t\right)\right)Wx\left(t-\tau \left(t\right)\right)\\ \kappa (0) & = & \kappa_{0} \end{array}\right. \end{array}$$

where $\kappa_{0}\geq 0$, ς is a function of class $K_{\infty }$ with Lipschitz continuous [24]. Compared with static event triggering scheme, dynamic event triggering scheme is less conservative that do not have to satisfy $\sigma x^{T}\left(t\right)Wx\left(t\right)-e^{T}\left(t\right)We\left(t\right)\geq 0$.

**Remark 1.**  *Notably, several factors that need to be carefully chosen for the communication strategy may be found in the event-triggered mechanism (16). Firstly, a bigger σ may accept a larger measured error e(t), which results in fewer data packets being triggered for the controller update. σ describes how tight the triggering process is. The internal dynamic variable κ(t) participation is then described by the parameter ρ.*

**Remark 2.**  *The internal variable κ(t) under the starting $\kappa_{0}\geq 0$ meets the following criterion for the dynamic event-triggered scheme (16)*

$$\begin{array}{c} \kappa (t)\geq 0,\;\rho >0. \end{array}$$

For the rest of this paper, we will consider the flexible manipulator subject to the semi-Markov jump process in the network environment, through the design of the corresponding event-trigger controller to make the system state asymptotically stable and meet the $H_{\infty }$ performance of parameter γ. The event-trigger controller is selected as follows:

(18) $$\begin{array}{c} u\left(t\right)=K_{i}\left(x\left(t-\tau \left(t\right)\right)-e\left(t\right)\right) \end{array}$$

**Remark 3.**  *Compared with the traditional static ETM, there are two advantages on the proposed DETM. First, the DETM can effectively avoid the transmission of redundant data and reduce the number of triggering compared with static ETM. Second, the event-triggered parameter ς(κ(t)) can be dynamically selected adaptively, which provides greater potential for optimization of the event-trigger mechanism. In this paper, the parameter ς(κ(t)) is assumed to be a direct proportional function which belongs to a function of class $K_{\infty }$ with Lipschitz continuous. Furthermore, more options for the event-trigger parameter can be seen in existing results [31,32].*

In the development of network technology, some hackers seek personal gains by launching network attacks, which represents both an immoral and illegal act. In this article, we consider a common form of network attack: DoS attacks. The Bernoulli process is used to describe the phenomena, the following is suggested for the affected controller:

(19) $$\begin{array}{c} \bar{u}\left(t\right)=h\left(t\right)u\left(t\right), \end{array}$$

where the Bernoulli distributed variables h(t) display the frequency of DoS assaults and $E\{h\left(t\right)=h_{1}\}$. Introduce into switch matrix *L* to describe DoS attacks:

$$\begin{array}{c} L=\left\{\begin{array}{cc} 1 & \mathrm{No}\;\mathrm{attack},\\ 0 & \mathrm{DoS}\;\mathrm{attack}, \end{array}\right. \end{array}$$

In summary, the manipulator’s closed-loop control system is stated as

(20) $$\begin{array}{c} \left\{\begin{array}{c} \dot{x}\left(t\right)=A_{i}x\left(t\right)+B_{1i}\omega \left(t\right)+B_{2}LK_{i}x\left(t-\tau \left(t\right)\right)-B_{2}LK_{i}e\left(t\right)\\ z\left(t\right)=Cx\left(t\right),\;t\in \left[t_{k}h+\tau_{t_{k}},t_{k+1}h+\tau_{t_{k+1}}\right]\\ x\left(t\right)=\vartheta \left(t\right),\;t\in \left[-\tau_{2},0\right] \end{array}\right. \end{array}$$

where ϑ(t) is the initial function of x(t).

Some necessary lemmas are presented to complete the following theorem.

**Problem Statement:**  *For a given semi-Markov flexible robotic arm system suffering from DoS attacks in (5), the dynamic event-triggered controller is designed in (19), so that the resulting system in (20) is asymptotically stable with a $H_{\infty }$ performance.*

**Lemma 2.**  *[33] For matrix R>0, scalar a,b, satisfy a<b. The function $x\left(s\right):\left[a,b\right]\to R^{n}$ is continuously differentiable, and the following integral inequality is true*

(21) $$\begin{array}{c} -\left(b-a\right)\int_{a}^{b}{\dot{x}}^{T}\left(s\right)R\dot{x}\left(s\right)ds\leq -{\left[\int_{a}^{b}\dot{x}\left(s\right)ds\right]}^{T}R\left[\int_{a}^{b}\dot{x}\left(s\right)ds\right] \end{array}$$

**Lemma 3.**  *[34] For a given scalar 0≤α≤1,the matrix $W_{1},W_{2}\in R^{n}\times m$, the positive matrix $R\in R^{n}$, if there is a matrix $N\in R^{n}$, satisfies $\left[\begin{array}{cc} S & N\\ {}* & S \end{array}\right]>0$, then the following inequality is true*

(22) $$\begin{array}{c} -\frac{1}{\alpha }W_{1}^{T}RW_{1}-\frac{1}{1-\alpha }W_{2}^{T}RW_{2}\leq -{\left[\begin{array}{c} W_{1}\\ W_{2} \end{array}\right]}^{T}\left[\begin{array}{cc} R & N\\ {}* & R \end{array}\right]\left[\begin{array}{c} W_{1}\\ W_{2} \end{array}\right] \end{array}$$

## 3. Main Results

### 3.1. Stability Analysis

In this section, a sufficient condition of asymptotically stable is presented for the proposed flexible robotic arm system with an event-triggered controller in (20).

**Theorem 1.**  *For given scalars $\tau_{1}$, $\tau_{2}$, σ∈(0,1), the closed-loop system in (20) is asymptotically stable with a $H_{\infty }$ performance γ if a matrix N can be found, in addition to positive definite symmetric matrices $P_{i}$, $Q_{1}$, $Q_{2}$, $R_{j}\left(j=1,2,3\right)$, $W_{i}\left(i=1,2,3,4\right)$ satisfying the following inequalities*

(23) $$\begin{array}{c} \Omega_{i}=\left[\begin{array}{ccc} \Omega_{1i} & \Omega_{2i} & \Omega_{3i}\\ {}* & \Omega_{4} & 0\\ {}* & * & \Omega_{5} \end{array}\right]-\varsigma \left(\kappa \left(t\right)\right)<0,\;i\in S. \end{array}$$

*where*

$$\begin{array}{ccc} \Omega_{1i} & = & \left[\begin{array}{cccc} \Phi_{11i} & P_{i}B_{2}LK_{i} & R_{1} & R_{2}\\ {}* & \Phi_{22} & R_{3}-N^{T} & R_{3}-N\\ {}* & * & \Phi_{33} & N\\ {}* & * & * & \Phi_{44} \end{array}\right],\\ \Omega_{2i} & = & \left[-P_{i}{\hat{B}}_{2}LK_{i}\;\;P_{i}{\hat{B}}_{1i}\;\;\hat{C}\;\right],\;{\hat{B}}_{2}={\left[\;B_{2}^{T}\;\;0_{n}\;\;0_{n}\;\;0_{n}\;\right]}^{T},\;{\hat{B}}_{1i}={\left[\;B_{1i}^{T}\;\;0_{n}\;\;0_{n}\;\;0_{n}\;\right]}^{T},\\ \Omega_{3i} & = & \left[\tau_{1}A_{i}^{T}\left(t\right)\;\;\tau_{2}A_{i}^{T}\left(t\right)\;\;\tau_{12}A_{i}^{T}\left(t\right)\right],\;\;\hat{C}={\left[\;C\;\;0_{n}\;\;0_{n}\;\;0_{n}\;\right]}^{T},\;\tau_{12}=\tau_{2}-\tau_{1},\\ \Omega_{4} & = & -\mathrm{diag}\left\{W,\gamma^{2}I,I\right\},\;\Omega_{5}=-\mathrm{diag}\left\{R_{1}^{-1},R_{2}^{-1},R_{3}^{-1}\right\},\;{\hat{P}}_{i}=\sum_{j=1}^{s}\pi_{ij}P_{j},\\ \Phi_{11i} & = & A_{i}^{T}P_{i}+P_{i}A_{i}+Q_{1}+Q_{2}-R_{1}-R_{2}+{\hat{P}}_{i},\;\Phi_{22}=N+N^{T}-2R_{3}+\sigma W,\\ \Phi_{33} & = & -R_{3}-R_{1}-Q_{1},\;\;\Phi_{44}=-R_{3}-R_{2}-Q_{2},\\ A_{i}\left(t\right) & = & [A_{r}\left(t\right)\;\;B_{2}LK_{i}\;\;0_{n}\;\;0_{n}\;\;-B_{2}LK_{i}\;\;B_{1i}]. \end{array}$$

**Proof.**  Let us build a Lyapunov–Krasovskii Functional (LKF) as follows:

(24) $$\begin{array}{c} V\left(x\left(t\right),r\left(t\right),\kappa \left(t\right)\right)=V_{1}\left(x\left(t\right),r\left(t\right)=i\right)+V_{2}\left(x\left(t\right)\right)+V_{3}\left(x\left(t\right)\right)+\kappa \left(t\right) \end{array}$$

where

$$\begin{array}{ccc} V_{1}\left(x\left(t\right),i\right) & = & x^{T}(\left(t\right)P_{i}x\left(t\right),\\ V_{2}\left(x\left(t\right)\right) & = & \int_{t-\tau_{1}}^{t}x^{T}\left(s\right)Q_{1}x\left(s\right)ds+\int_{t-\tau_{2}}^{t}x^{T}\left(s\right)Q_{2}x\left(s\right)ds\\ V_{3}\left(x\left(t\right)\right) & = & \tau_{1}\int_{-\tau_{1}}^{0}\int_{t+\theta }^{t}{\dot{x}}^{T}\left(s\right)R_{1}\dot{x}\left(s\right)dsd\theta \\ & & +\tau_{2}\int_{-\tau_{2}}^{0}\int_{t+\theta }^{t}{\dot{x}}^{T}\left(s\right)R_{2}\dot{x}\left(s\right)dsd\theta \\ & & +\tau_{12}\int_{-\tau_{2}}^{-\tau_{1}}\int_{t+\theta }^{t}{\dot{x}}^{T}\left(s\right)R_{3}\dot{x}\left(s\right)dsd\theta \end{array}$$

Taking the derivative of $t\in \left[t_{k}+\tau_{t_{k}},t_{k+1}+\tau_{t_{k+1}}\right)$ along the system track, we get

(25) $$\begin{array}{ccc} LV_{1}\left(x\left(t\right),i\right) & = & \lim_{△\to 0}\frac{E\{V(x(t+∆),r(t+∆)\}-V(x,i)}{△}\\ & = & {\dot{x}}^{T}\left(t\right)P_{i}x\left(t\right)+x^{T}\left(t\right)P_{i}\dot{x}\left(t\right)+x^{T}\left(t\right){\hat{P}}_{i}x\left(t\right) \end{array}$$

where ${\hat{P}}_{i}=\sum_{j=1}^{s}\pi_{ij}P_{j}$, and

(26) $$\begin{array}{ccc} {\dot{V}}_{2}\left(x\left(t\right)\right) & = & x^{T}\left(t\right)Q_{1}x\left(t\right)-x^{T}\left(t-\tau_{1}\right)Q_{1}x\left(t-\tau_{1}\right)\\ & & +x^{T}\left(t\right)Q_{2}x\left(t\right)-x^{T}\left(t-\tau_{2}\right)Q_{2}x\left(t-\tau_{2}\right)\\ {\dot{V}}_{3}\left(x\left(t\right)\right) & = & {\dot{x}}^{T}\left(t\right)\bar{R}\dot{x}\left(t\right)-\tau_{1}\int_{t-\tau_{1}}^{t}{\dot{x}}^{T}\left(s\right)R_{1}\dot{x}\left(s\right)ds\\ & & -\tau_{2}\int_{t-\tau_{2}}^{t}{\dot{x}}^{T}\left(s\right)R_{2}\dot{x}\left(s\right)ds-\tau_{12}\int_{t-\tau_{2}}^{t-\tau_{1}}{\dot{x}}^{T}\left(s\right)R_{3}\dot{x}\left(s\right)ds \end{array}$$

(27) $$\begin{array}{ccc} \dot{\kappa }\left(t\right) & = & -\varsigma \left(\kappa \left(t\right)\right)-e^{T}\left(t\right)We\left(t\right)+\sigma x^{T}\left(t-\tau \left(t\right)\right)WX\left(t-\tau \left(t\right)\right) \end{array}$$

where $\bar{R}=\tau_{1}^{2}R_{1}+\tau_{2}^{2}R_{2}+\tau_{12}^{2}R_{3}$. If we define $\xi \left(t\right)={\left[\;x^{T}\left(t\right)\;\;x^{T}\left(t-\tau \left(t\right)\right)\;\;x^{T}\left(t-\tau_{1}\right)\;\;x^{T}\left(t-\tau_{2}\right)\;\;e^{T}\left(t\right)\;\;\omega^{T}\left(t\right)\;\right]}^{T}$, the $\dot{x}\left(t\right)=A_{i}\xi \left(t\right)$ satisfies $A_{i}\left(t\right)=\left[\;A_{r}\left(t\right)\;\;B_{2}LK_{i}\;\;0_{n}\;\;0_{n}\;\;-B_{2}K_{i}\;\;B_{1i}\;\right]$.

Using Lemmas 2 and 3, we can obtain the following inequalities with l=1,2:

(28) $$\begin{array}{ccc} -\tau_{l}\int_{t-\tau_{l}}^{t}{\dot{x}}^{T}\left(s\right)R_{l}\dot{x}\left(s\right)ds & \leq & -G_{l}^{T}\left[\begin{array}{cc} R_{l} & -R_{l}\\ {}* & R_{l} \end{array}\right]G_{l} \end{array}$$

(29) $$\begin{array}{ccc} -\tau_{12}\int_{t-\tau_{2}}^{t-\tau_{1}}{\dot{x}}^{T}\left(s\right)R_{3}\dot{x}\left(s\right)ds & \leq & -G_{3}^{T}\left[\begin{array}{cc} R_{3} & N\\ {}* & R_{3} \end{array}\right]G_{3} \end{array}$$

where $G_{l}={\left[\;x^{T}\left(t\right)\;\;x^{T}\left(t-\tau_{l}\right)\;\right]}^{T}$, (l=1,2), $G_{3}={\left[\;x^{T}\left(t-\tau_{1}\right)-x^{T}\left(t-\tau \left(t\right)\right)\;\;x^{T}\left(t-\tau \left(t\right)\right)-x^{T}\left(t-\tau_{2}\right)\;\right]}^{T}$.

Combining (25)–(29) with the event-triggered condition in (16), we have

(30) $$\begin{array}{ccc} LV(x(t),r(t),k(t)) & = & \xi^{T}\left(t\right)\Upsilon_{1}\xi \left(t\right)+\xi^{T}\left(t\right)\Upsilon_{2}\xi \left(t\right)+\xi^{T}\left(t\right)\Upsilon_{3}\xi \left(t\right)-\varsigma \left(\kappa \left(t\right)\right) \end{array}$$

where

$$\begin{array}{ccc} \Upsilon_{1} & = & \left[\begin{array}{cc} \Upsilon_{111} & \Upsilon_{112}\\ {}* & 0_{5\times 5} \end{array}\right],\;\Upsilon_{111}=A_{i}^{T}P_{i}+P_{i}A_{i}+{\hat{P}}_{i},\\ \Upsilon_{112} & = & \left[\begin{array}{ccccc} P_{i}B_{2}LK_{i} & 0_{n} & 0_{n} & -P_{i}B_{2}LK_{i} & P_{i}B_{1i} \end{array}\right],\\ \Upsilon_{2} & = & \left[\begin{array}{cc} \Upsilon_{211} & 0_{2\times 4}\\ {}* & \Upsilon_{222} \end{array}\right],\;\Upsilon_{211}=\left[\begin{array}{cc} Q_{1}+Q_{2} & 0\\ {}* & 0 \end{array}\right],\\ \Upsilon_{222} & = & \left[\begin{array}{ccc} -Q_{1} & 0 & 0_{1\times 2}\\ {}* & -Q_{2} & 0_{1\times 2}\\ {}* & * & 0_{2\times 2} \end{array}\right],\;\Upsilon_{3}=A_{i}^{T}\bar{R}A_{i}+\left[\begin{array}{cc} \Upsilon_{311} & 0_{4\times 2}\\ {}* & 0_{2\times 2} \end{array}\right],\\ \Upsilon_{311} & = & \left[\begin{array}{cccc} -R_{1}-R_{2} & 0 & R_{1} & R_{2}\\ {}* & N+N^{T}-2R_{3} & R_{3}-N^{T} & R_{3}-N\\ {}* & * & -R_{1}-R_{3} & N\\ {}* & * & * & -R_{2}-R_{3} \end{array}\right]. \end{array}$$

where $\bar{R}$ and $A_{i}$ are defined in (23).

For the $H_{\infty }$ performance, let us define

$$\begin{array}{c} J_{T}\left(x\left(t\right),r\left(t\right)\right)≜E\left\{\int_{0}^{T}\left[z^{T}\left(t\right)z\left(t\right)-\gamma^{2}w^{T}\left(t\right)w\left(t\right)\right]dt\right\}, \end{array}$$

under the zero initial condition $\nu_{0}=0$ for $t\in [-\tau_{2},0]$. Using the Dynkin’s citation formula mentioned in [35] and $V(\nu_{0},r_{0})=0$, we have

$$\begin{array}{c} E\{V\left(x\left(T\right),r\left(T\right)\right\}=E\left\{\int_{0}^{T}LV\left(x\left(s\right),r\left(s\right)\right)ds\right\} \end{array}$$

the following statement is true for any nonzero $\omega \left(t\right)\in L_{2}\left[0,\infty \right)$:

$$\begin{array}{ccc} J_{T} & = & E\left\{\int_{0}^{T}\left[z^{T}\left(t\right)z\left(t\right)-\gamma^{2}\omega^{T}\left(t\right)\omega \left(t\right)\right]dt\right\}\\ & = & E\left\{\int_{0}^{T}\left[z^{T}\left(t\right)z\left(t\right)-\gamma^{2}\omega^{T}\left(t\right)\omega \left(t\right)\right]dt+LV\left(x\left(t\right),r\left(t\right)\right)\right\}-E\left\{V\left(x\left(T\right),r\left(T\right)\right)\right\}\\ & \leq & E\left\{\int_{0}^{T}\left[z^{T}\left(t\right)z\left(t\right)-\gamma^{2}w^{T}\left(t\right)w\left(t\right)+LV\left(x\left(t\right),r\left(t\right)\right)\right]dt\right\}\\ & = & E\left\{\int_{0}^{T}\left(\xi^{T}\left(t\right)\left(\Upsilon_{1}+\Upsilon_{2}+\Upsilon_{3}\right)\xi \left(t\right)+z^{T}\left(t\right)z\left(t\right)-\gamma^{2}w^{T}\left(t\right)w\left(t\right)-\varsigma \left(\kappa \left(t\right)\right)\right)dt\right\}. \end{array}$$

according to Schur complement lemma, $\xi^{T}\left(t\right)\left(\Upsilon_{1}+\Upsilon_{2}+\Upsilon_{3}\right)\xi \left(t\right)+z^{T}\left(t\right)z\left(t\right)-\gamma^{2}w^{T}\left(t\right)w\left(t\right)-\varsigma \left(\kappa \left(t\right)\right)$ can be equivalent to (23). If $\Omega_{i}<0$, then

$$\begin{array}{c} LV\left(x\left(t\right),r\left(t\right)\right)\leq -z^{T}\left(t\right)z\left(t\right)+\gamma^{2}\omega^{T}\left(t\right)\omega \left(t\right) \end{array}$$

integrate both sides of equation from $\left[t_{k}h+\tau_{t_{k}},t_{k+1}h+\tau_{t_{k+1}}\right)$, where ${⋃}_{k=1}^{\infty }\left[t_{k}h+\tau_{t_{k}},t_{k+1}h+\tau_{t_{k+1}}\right]=\left[t_{0},\infty \right)$, then $LV\left(x\left(\infty \right),r\left(\infty \right)\right)-LV\left(x\left(t_{0}\right),r\left(t_{0}\right)\right)\leq \int_{t_{0}}^{\infty }\left[-z^{T}\left(s\right)z\left(s\right)-\gamma^{2}\omega^{T}\left(s\right)\omega \left(s\right)\right]ds$ satisfies under zero initial condition, namely ${∥z\left(t\right)∥}_{2}\leq \gamma {∥\omega \left(t\right)∥}_{2}$. The proof is finished. □

**Remark 4.**  *In this section, the delay-dependent condition based on the LKF is construct. This is our first attempt to apply the stochastic theory and LMI technology directly to the control of flexible robotic arm systems in network communication. The LKF method has a more mature theoretical system and has been proved to be feasible. Furthermore, this method also can be easily extended to dissipative analysis, exponential stability and so on.*

### 3.2. Co-Design of the Event-Triggered and Controller Parameters

In this section, the state controller of the proposed flexible robotic arm system using dynamic event-triggered scheme is presented in (20).

**Theorem 2.**  *For given scalars $\tau_{1}$, $\tau_{2}$, ϖ, σ∈(0,1) and $\varphi_{j}\left(j=1,2,3,4\right)$, if there exist matrices N, and positive definite symmetric matrices $Y_{i}$, ${\tilde{Q}}_{1}$, ${\tilde{Q}}_{2}$, ${\tilde{R}}_{j}\left(j=1,2,3\right)$, $W_{i}$, $X_{i}\left(i=1,2,3,4\right)$, scalar γ>0 satisfying the following inequalities*

(31) $$\begin{array}{ccc} \Pi_{i} & = & \left[\begin{array}{cccc} \Pi_{11i} & \Pi_{12i} & \Pi_{13i} & \Pi_{14i}\\ {}* & \Pi_{22i} & 0 & 0\\ {}* & * & \Pi_{33i} & 0\\ {}* & * & * & \Pi_{44i} \end{array}\right]<0,\;i\in S \end{array}$$

*where*

$$\begin{array}{ccc} \Pi_{11i} & = & \left[\begin{array}{cccc} \Gamma_{11} & B_{2}Y_{i} & {\tilde{R}}_{1} & {\tilde{R}}_{2}\\ {}* & \Gamma_{22} & {\tilde{R}}_{3}-{\tilde{N}}^{T} & {\tilde{R}}_{3}-\tilde{N}\\ {}* & * & \Gamma_{33} & \tilde{N}\\ {}* & * & * & \Gamma_{44} \end{array}\right],\;\Pi_{12i}=\left[-{\hat{B}}_{2}LY_{i}\;\;{\hat{B}}_{1i}\;\;X_{i}\hat{C}\;\right],\\ \Pi_{13i} & = & \left[\tau_{1}{\tilde{A}}_{i}^{T}\left(t\right)\;\;\tau_{2}{\tilde{A}}_{i}^{T}\left(t\right)\;\;\tau_{12}{\tilde{A}}_{i}^{T}\left(t\right)\right],\;\;\Pi_{22i}=-\mathrm{diag}\left\{2\varphi_{4}X_{i}-\varphi_{4}^{2}W,\gamma^{2}I,I\right\},\\ \Pi_{33i} & = & \mathrm{diag}\left\{\varphi_{1}^{2}{\tilde{R}}_{1}-2\varphi_{1}X_{i},\varphi_{2}^{2}{\tilde{R}}_{2}-2\varphi_{2}X_{i},\varphi_{3}^{2}{\tilde{R}}_{3}-2\varphi_{3}X_{i}\right\},\\ \Pi_{14i} & = & [\sqrt{\pi_{i,1}}X_{i}\;\;\cdots \;\sqrt{\pi_{i,i-1}}X_{i}\;\;\sqrt{\pi_{i,i+1}}X_{i}\;\cdots \;\sqrt{\pi_{i,s}}X_{i}\;\;X_{i}],\\ \Pi_{44i} & = & -\mathrm{diag}\left\{X_{1},\cdots ,X_{i-1},X_{i+1},\cdots ,X_{s},\varpi^{-1}I\right\}\\ \Gamma_{11} & = & X_{i}A_{i}^{T}+A_{i}X_{i}+{\tilde{Q}}_{1}+{\tilde{Q}}_{2}-{\tilde{R}}_{1}-{\tilde{R}}_{2}+\pi_{ii}X_{i},\\ \Gamma_{22} & = & \tilde{N}+{\tilde{N}}^{T}-2{\tilde{R}}_{3}+2\sigma \varphi_{4}X_{i}-\sigma \varphi_{4}^{2}W,\\ \Gamma_{33} & = & -{\tilde{R}}_{3}-{\tilde{R}}_{1}-{\tilde{Q}}_{1},\;\Gamma_{44}=-{\tilde{R}}_{3}-{\tilde{R}}_{2}-{\tilde{Q}}_{2},\\ {\tilde{A}}_{i}\left(t\right) & = & [\;A_{r}\left(t\right)X_{i}\;\;B_{2}LY_{i}\;\;0_{n}\;\;0_{n}\;\;-B_{2}LY_{i}\;\;B_{1i}\;], \end{array}$$

*and $\tilde{Y}=XYX,\;Y\in \left\{N,N_{T},Q_{1},Q_{2},R_{i}\right\}\left(i=1,2,3\right)$; ${\hat{B}}_{2}$, ${\hat{B}}_{1i}$, $\hat{C}$, $\tau_{12}$ are defined in Theorem 23, and then the closed loop system in (20) with the event-triggered controller in (18) is asymptotically stable with a $H_{\infty }$ performance γ, namely ${∥z\left(t\right)∥}_{2}\leq \gamma {∥\omega \left(t\right)∥}_{2}$, the corresponding controller is $K_{i}=Y_{i}X_{i}^{-1}$.*

**Proof.**  Choose an LKF V(x,i,κ) as in (24). Note that when the external disturbance ω(t)=0, according to Theorem 1, we can get LV(x,i,κ)<0 naturally, this shows the asymptotic stability of the closed-loop system. ς(κ(t)) is a function of class $K_{\infty }$, $\varsigma \left(\kappa \left(t\right)\right)=\varpi \kappa \left(t\right),\varpi \in R^{0+}$ clearly satisfies this requirement.

(32) $$\begin{array}{c} {\tilde{\Omega }}_{i}=\left[\begin{array}{cccc} \Omega_{1i} & \Omega_{2i} & \Omega_{3i} & I\\ {}* & \Omega_{4} & 0 & 0\\ {}* & * & \Omega_{5} & 0\\ {}* & * & * & \varpi^{-1}I \end{array}\right]<0,\;i\in S. \end{array}$$

According to Theorem 1, let is assume that $X_{i}=P_{i}^{-1},Y_{i}=K_{i}X_{i}$(i∈S). Then, multiply both sides of this inequality by $\left\{X_{i},X_{i},X_{i},X_{i},X_{i},I,I,I,I,I,I\right\}$

$$\begin{array}{ccc} {\hat{\Omega }}_{i} & = & \left[\begin{array}{cccc} {\hat{\Pi }}_{11i} & \Pi_{12i} & \Pi_{13i} & X_{i}\\ {}* & {\hat{\Pi }}_{22i} & 0 & 0\\ {}* & * & {\hat{\Pi }}_{33i} & 0\\ {}* & * & * & \varpi^{-1}I \end{array}\right]<0,\;i\in S \end{array}$$

where

$$\begin{array}{ccc} {\hat{\Pi }}_{11i} & = & \left[\begin{array}{cccc} {\hat{\Gamma }}_{11} & B_{2}LY_{i} & {\tilde{R}}_{1} & {\tilde{R}}_{2}\\ {}* & \Gamma_{22} & {\tilde{R}}_{3}-{\tilde{N}}^{T} & {\tilde{R}}_{3}-\tilde{N}\\ {}* & * & \Gamma_{33} & \tilde{N}\\ {}* & * & * & \Gamma_{44} \end{array}\right],\\ {\hat{\Gamma }}_{11} & = & A_{i}^{T}X_{i}+X_{i}A_{i}-{\tilde{R}}_{1}-{\tilde{R}}_{2}+{\tilde{Q}}_{1}+{\tilde{Q}}_{2}+\pi_{ii}X_{i}+\sum_{i\neq j,j=1}^{s}\pi_{ij}X_{i}X_{j}^{-1}X_{i},\\ {\hat{\Pi }}_{33i} & = & -\mathrm{diag}\left(X_{i}{\left(X_{i}R_{1}X_{i}\right)}^{-1}X_{i},X_{i}{\left(X_{i}R_{2}X_{i}\right)}^{-1}X_{i},X_{i}{\left(X_{i}R_{3}X_{i}\right)}^{-1}X_{i}\right) \end{array}$$

There exists $-X_{i}{\tilde{R}}_{j}^{-1}X_{i}$(j=1,2,3) nonlinear term in the equation, which is linearized by inequation $-X_{i}{\tilde{R}}_{j}^{-1}X_{i}<\varphi_{j}^{2}{\tilde{R}}_{j}-2\varphi_{j}X_{i}$(j=1,2,3.i∈S). In order to implement in the LMI toolbox of MATLAB, according to Schur complement lemma ${\hat{\Omega }}_{i}<0$ is further restructured to (31). □

**Remark 5.**  *Under the condition of $\varsigma \left(\kappa \left(t\right)\right)=\varpi \kappa \left(t\right),\varpi \in R^{0+}$, the inter-event intervals always have a positive lower bound, which rules out Zeno behavior. Furthermore, because the internal dynamic variable κ(t) is not negative, the dynamic event-triggered technique utilized might result in less data transfer, which helps conserve resources.*

The implementation procedure of the dynamic event-triggered robust controller design for flexible robotic arm systems with continuous-time phase-type semi-Markov jump process is illustrated in Algorithm 1.

**Algorithm 1** Dynamic Event-triggered Robust Controller DesignPhysical constants: *M*, *g*, *H*, *k*, *J*;Random variables: $I_{i}$, $h_{1}$;**Initialization:** (For each state *i*)1. Initialize Initial state ϑ(0);2. Choose the lower and upper time delay in network $\tau_{1}$ and $\tau_{2}$;3. Choose the event-triggered parameters ϖ and σ;4. γ is the $H_{\infty }$ performance index;5. Set $P_{i}$, $Q_{1}$ $Q_{2}$, R1, R2 and R3 as positive definite matrices;**Iteration**:6. Let the iteration and step index s=0;7. Repeat8. The dynamic event-triggered control u(t)← (18)9.    **IF** event-triggered condition (16) is satisfied10.       not trigger, $u\left(t\right)=x\left(t_{k}h\right)$;11.       let s=s+1;11.    **ELSE**12.       update the signal $u\left(t\right)=x\left(t_{l}\right)$;13.       let s=s+1, $t_{k}h=t_{l}$;14.    **END IF**15.    **IF** exist DoS attacks;16.       let u(t)=0;17.    **ELSE**18.       normal signal transmission u(t);19.    **END IF**20. Go to back step (8);21. End.

## 4. Simulation Results

**Figure 5:**
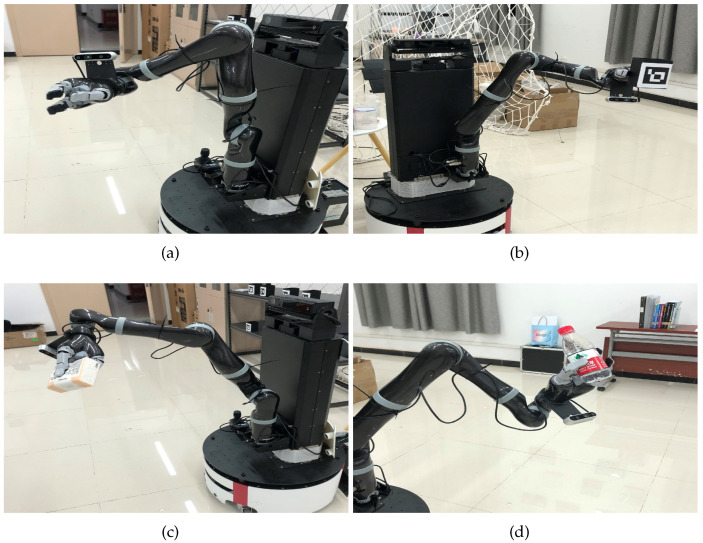
Application scenarios. (**a**) Model 1; (**b**) Model 2; (**c**) Model 3; (**d**) Model 4.

The flexible robotic arm in (20) shown grasping objects of different weights is very common in production practice. In this section, a phase-type semi-Markov chain is used to simulate the grabbing of four different objects as shown in Figure 5.

The sojourn time in the first three states is a random negative exponential distribution variable of parameter $\lambda_{j}\;\left(j=1,2,3\right)$. The sojourn time in the last state is split into two parts, namely two random variables with parameters $\lambda_{4}$ and $\lambda_{4}$ of negative exponential distribution. For example, if the process $\hat{r}\left(t\right)$ enters a functioning condition, it must first spend some time in the first part, then stay in the second part, and then enter state 1 at the end. Without loss of generality, we assume that

$$\begin{array}{ccc} P & = & \left[\begin{array}{cccc} p_{11} & p_{12} & p_{13} & p_{14}\\ p_{21} & p_{22} & p_{23} & p_{24}\\ p_{31} & p_{32} & p_{33} & p_{34}\\ p_{41} & p_{42} & p_{43} & p_{44} \end{array}\right]=\left[\begin{array}{cccc} 0 & 0.3 & 0.5 & 0.2\\ 0.3 & 0 & 0.6 & 0.1\\ 0.3 & 0.2 & 0 & 0.5\\ 0.1 & 0.4 & 0.5 & 0 \end{array}\right],\\ a^{j} & = & \left(a_{1}^{j}\right)=\left(1\right),\;T^{j}=\left(T_{11}^{j}\right)=\left(-\lambda_{j}\right)\left(j=1,2,3\right),\\ a^{4} & = & \left(a_{1}^{4},a_{2}^{4}\right)=\left[1,0\right],\;T^{4}=\left[\begin{array}{cc} T_{11}^{4} & T_{12}^{4}\\ T_{21}^{4} & T_{22}^{4} \end{array}\right]=\left[\begin{array}{cc} -\lambda_{4} & \lambda_{4}\\ 0 & \lambda_{5} \end{array}\right]. \end{array}$$

The state space of $Z\left(t\right)=(\hat{r}\left(t\right),\chi \left(t\right))$ is clearly U=((1,1),(2,1),(3,1),(4,1),(4,2)). We order each component of U as follows:

$$\begin{array}{c} \psi ((1,1))=1,\;\psi ((2,1))=2,\;\psi ((3,1))=3,\;\psi ((4,1))=4,\;\psi ((4,2))=5, \end{array}$$

as a result, the infinitesimal generator of ψ(Z(t)) is

(33) $$\begin{array}{c} \Xi =\left[\begin{array}{ccccc} -\lambda_{1} & 0.3\lambda_{1} & 0.5\lambda_{1} & 0.2\lambda_{1} & 0\\ 0.3\lambda_{2} & -\lambda_{2} & 0.6\lambda_{2} & 0.1\lambda_{2} & 0\\ 0.3\lambda_{3} & 0.2\lambda_{3} & -\lambda_{3} & 0.5\lambda_{3} & 0\\ 0 & 0 & 0 & -\lambda_{4} & \lambda_{4}\\ 0.1\lambda_{5} & 0.4\lambda_{5} & 0.5\lambda_{5} & 0 & -\lambda_{5} \end{array}\right]. \end{array}$$

Before presenting the result of the numerical simulation, the setup and software of the computer that we employ are given in Table 1:

Similar to [17], some appropriate physical system parameters of (5) and parameters related to the dynamic event-trigger condition in (16) are set in the following Table 2.

Let us choose $\vartheta \left(t\right)={\left[-0.3\;0.4\;0.6\;-0.8\right]}^{T}$, $t\in \left[-\tau_{2},0\right]$, and select 30 s as the total simulation time, and assume that the external disturbance is w(t)=exp(−t)sin(t). The dynamic event-triggered weighting matrix $W_{i}$ and the corresponding controller $K_{i}\left(i=1,2,3,4\right)$ are solved by the LMI program and given as follows:

$$\begin{array}{ccc} W_{1} & = & \left[\begin{array}{cccc} 32.436 & 1.5572 & 32.527 & 1.7520\\ 1.5572 & 76.951 & 0.20833 & -1.8370\\ 32.527 & 0.20833 & 48.101 & 1.6751\\ 1.7520 & -1.8370 & 1.6751 & 0.35863 \end{array}\right]\times 10^{-10},\;K_{1}={\left[\begin{array}{c} 0.0863\\ -0.0347\\ -0.0115\\ -1.3406 \end{array}\right]}^{T},\\ W_{2} & = & \left[\begin{array}{cccc} 34.138 & 0.5318 & 34.187 & -0.0194\\ 0.5318 & 67.814 & -0.1582 & -0.3244\\ 34.187 & -0.1582 & 50.187 & 0.2248\\ -0.0194 & -0.3244 & 0.2248 & 0.0639 \end{array}\right]\times 10^{-10},\;K_{2}={\left[\begin{array}{c} -0.0219\\ 0.0060\\ 0.0210\\ -1.3341 \end{array}\right]}^{T},\\ W_{3} & = & \left[\begin{array}{cccc} 33.091 & 1.2368 & 33.154 & 1.0846\\ 1.2368 & 73.571 & -0.1185 & -1.3694\\ 33.154 & -0.1185 & 48.938 & 1.1624\\ 1.0846 & -1.3694 & 1.1624 & 0.3489 \end{array}\right]\times 10^{-10},\;K_{3}={\left[\begin{array}{c} 0.0414\\ -0.0267\\ 0.0039\\ -1.3394 \end{array}\right]}^{T},\\ W_{4} & = & \left[\begin{array}{cccc} 36.478 & -1.2711 & 36.377 & -2.8002\\ -1.2711 & 55.584 & -1.1178 & 2.6714\\ 36.377 & -1.1178 & 52.841 & -2.0251\\ -2.8002 & 2.6714 & -2.0251 & 0.8078 \end{array}\right]\times 10^{-10},\;K_{4}={\left[\begin{array}{c} -0.1647\\ 0.0606\\ 0.0637\\ -1.3346 \end{array}\right]}^{T}. \end{array}$$

**Figure 6:**
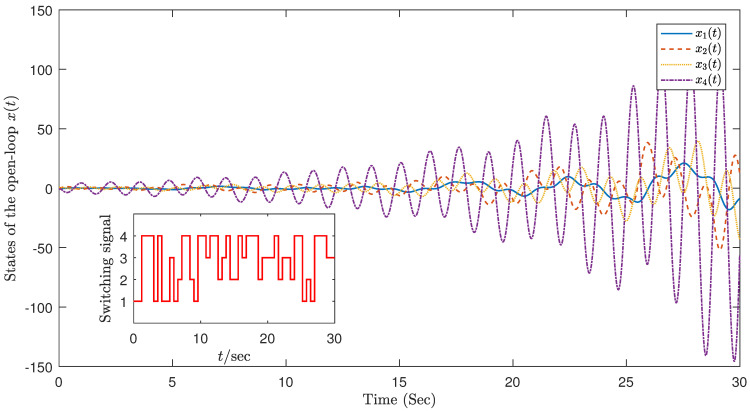
System status without controller.

Figure 6 displays the state-response diagram of the system under semi-Markov process without controller and shows that the open loop of the flexible robotic arm in (20) is unstable.

**Figure 7:**
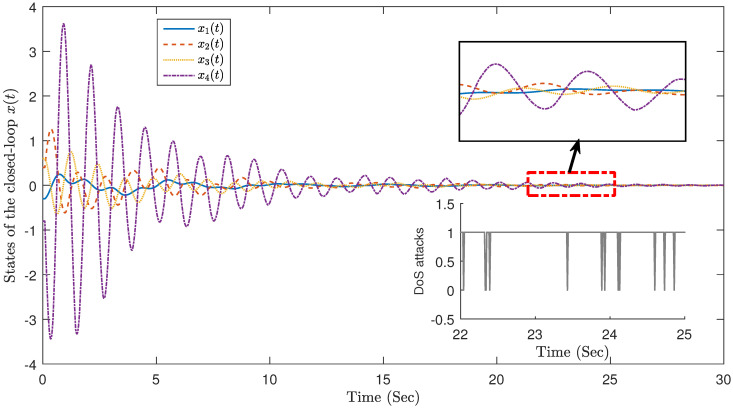
Event triggering mechanism.

The state trajectory of the static event-triggered mechanism is shown in Figure 7. Furthermore, DoS attacks that obey the Bernoulli distribution of parameter property $h_{1}=0.95$ operate on the input signal $u_{t}$. Contrapositive with the time-triggered mode, the event-triggered mode achieves fewer times of sending at the expense of a part of the calming time, a total of 193 events are triggered. Compared with the time period sampling 3000 times, the data transmission rate of the event trigger is 6.43%, which greatly saves network resources.

**Figure 8:**
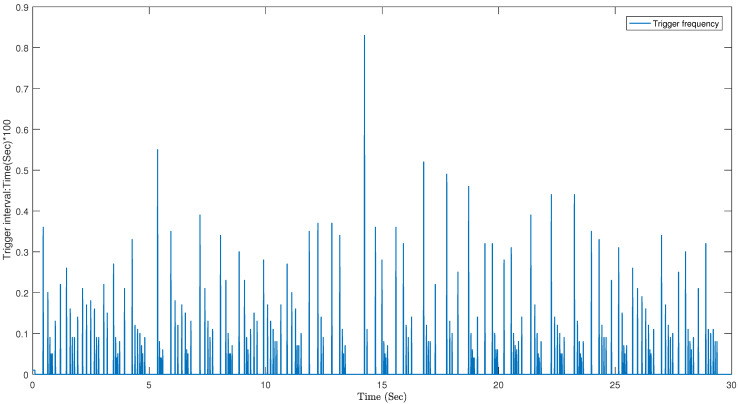
Trigger time interval (193 times).

Figure 8 depicts the interval between the sampling time when the control system successfully triggered under the event triggering mechanism and the last two successful triggering moments. As can be seen from the figure, the trigger intervals under the event triggering mechanism are not equal, and all trigger intervals are greater than or equal to the sampling cycle. Note Event triggering can reduce the transmission frequency of system data and save limited resources.

**Figure 9:**
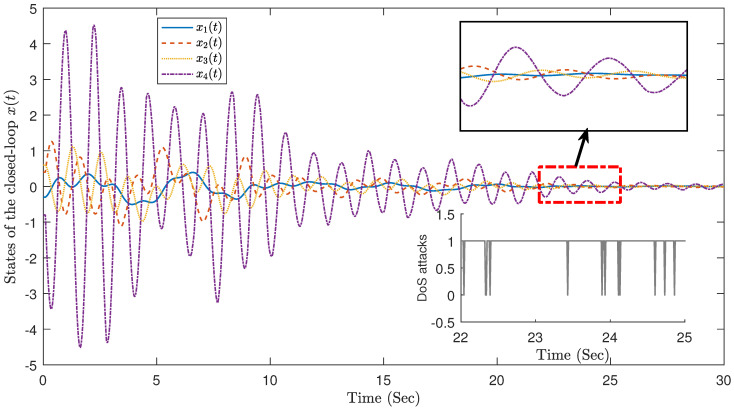
Dynamic event-triggering mechanism.

**Figure 10:**
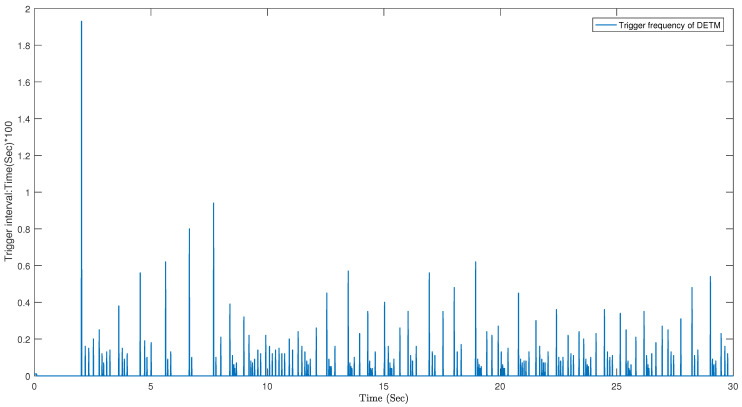
Dynamic trigger time interval (170 times).

The system state controlled by the dynamic event triggering mechanism is shown in Figure 9, and the matching triggering interval is shown in Figure 10. It has fewer trigger times (170, 5.67%) and exhibits a good calming effect for a limited time period. This demonstrates the effectiveness of the designed dynamic event-triggering controller in saving network resources.

In general, the proposed dynamic ETM can maintain the security and stability control of the flexible robotic arm systems while occupying far less network resources than the time-triggered mechanism and the static ETM. The minimum time-interval between two adjacent trigger instants is not less than the sampling period, to prevent the Zeno phenomenon. Furthermore, the threshold is dynamic, and thus the trigger condition can adaptively modify the transmission of the signal to fend off the negative effects of DoS attacks.

## 5. Conclusions

We herein present a type of physical model of flexible robot arm based on a semi-Markov jump process. For the corresponding linear system, the network control method is adopted, and the stabilization effect under the time-triggered mechanism and the dynamic event-triggered mechanism is considered, respectively. In order to obtain a full-state feedback controller satisfying the attenuation index γ of disturbance suppression, a series of matrix inequalities are obtained by constructing LKF and using Jensen inequality, reciprocal convex lemma and Schur complement lemma. The appropriate controller $K_{i}$ and weighted matrix $W_{i}$ are solved using the LMI toolkit in MATLAB. Finally, an effect diagram of the numerical simulation is given.

In this paper, for the first time, we take stochastic theory and LMI technologies into consideration in the control method of flexible robotic arm systems, and directly solve the controller parameters using LKF. However, this method still has some limitations. The angle q(θ) of the robot arm is assumed to be $\left(-\frac{\pi }{2},\frac{\pi }{2}\right)$, which is somewhat conservative. To extend the rotation of the arm to (0,2π), a neural-network-based approach will be considered in the future work. 

## Figures and Tables

**Table 1:** Simulation environment.

Hardware	Type
CPU	Intel(Core) i5-12400F
GPU	NVIDIA T400 4GB GDDR6
Memory stick	Kingston FURY 16GB
Hard disk	Kingston 1TB SSD
Software	Type
Operating system	Windows 10
MATLAB	Matlab 2016b

**Table 2:** Parameters of the controlled object.

Parameter	Unit	Numerical Value
$I_{1,\cdots ,4}$	$\;\mathrm{kg}\cdot m^{2}$	[1, 10]
*k*	$\;N\cdot m\cdot {\mathrm{rad}}^{-1}$	20
*J*	$\;\mathrm{kg}\cdot m^{2}$	1
$\tau_{1}$	s	0.01
*M*	kg	5
$\tau_{2}$	s	0.1
*H*	m	1
σ	*	0.6
ϖ	*	2
$h_{1}$	*	0.95

## Data Availability

The source codes and datasets used to support the findings of this study are available from the authors upon request via email: huiyanzhang@ctbu.edu.cn.

## Abbreviations

The following abbreviations are used in this manuscript:

MJSs	Markov Jump systems
PH	Phase-Type
NCSs	Networked Control Systems
DoS	Denial of Service
LMI	Linear Matrix Inequality
ETM	Event-triggered Mechanism
DETM	Dynamic Event-triggered Mechanism
LKF	Lyapunov–Krasovskii Functional
